# The effect of camel milk on house dust mite allergen induced asthma model in BALB/C mice

**DOI:** 10.1371/journal.pone.0327504

**Published:** 2025-06-27

**Authors:** Ayaulym Rakhmatulina, Shynar Kenenbay, Altynay Abuova, Maigul Kizatova, Akniyet Ibraikhan, Farrukh Makhmudov, Aitugan Mukashev, Aigerim Aitbaeva, Zhastalap Abilkaiyr, Galiya Ibadullayeva, Urishbay Chomanov, Akhmet Murzabulatov, Sanavar Azimova, Altyn Kulpiisova, Svetlana Bayantassova, Nurbek Aralbayev, Nurbibi Imanbayeva, Fatima Dikhanbayeva, Nadezhda Burambayeva, Nazgul Smagulova, Arman Issimov, Peter White

**Affiliations:** 1 Department of Mechanical Engineering and Robotics, Joldasbekov Institute of Mechanics and Engineering, Almaty, Kazakhstan; 2 Department of Food Technology, Food Safety and Quality, Almaty Technological University, Almaty, Kazakhstan; 3 Department of Food Technology, International Engineering Technological University, Almaty, Kazakhstan; 4 Department of Pharmaceutical Technology, S.D. Asfendiyarov Kazakh National Medical University, Almaty, Kazakhstan; 5 Kazakh-German Research Institute “Qymyz”, Almaty, Kazakhstan; 6 Department of Food Technology and Safety of Food Products, M. Auezov South – Kazakhstan University, Shymkent, Kazakhstan; 7 Department of Mechanical Engineering, Satbayev University, Almaty, Kazakhstan; 8 Scientific Consultant, “Paritet” LLP, Almaty, Kazakhstan; 9 Department of Veterinary Medicine, A. Baitursynov Kostanay Regional University, Kazakhstan; 10 Department of Veterinary Medicine, Zhangir Khan West Kazakhstan Agrarian Technical University, Oral, Kazakhstan; 11 Department of Zootechnology, Genetics and Breeding, Toraighyrov University, Pavlodar, Kazakhstan; 12 Department of Biology, Zhubanov Aktobe Regional University, Aktobe, Kazakhstan; 13 Sydney School of Veterinary Science, Faculty of Science, University of Sydney, Sydney, Australia; Yantai Institute of Technology, CHINA

## Abstract

Camel milk has demonstrated robust immunomodulatory and anti-inflammatory properties in various clinical and experimental studies. However, no previous studies have characterized the cellular immunological effects of camel milk in the context of allergic asthma. Therefore, the present work aimed to evaluate the protective effects of camel milk in house dust mite induced asthma in mice, which emulate human pulmonary inflammation. Female BALB/c mice aged 8- to 10-week-old were intranasally sensitized with vehicle or HDM in 2.5 µl (5 µg) per nostril, 5 days a week for 3 weeks. On day 22, mice received an HDM challenge by a large volume but low dose into the lung (5 µg in 50µl) using intranasal inoculation. Using oral gavage technique, CM/HDM group mice received 0.5 ml of camel milk or vehicle five times a week, starting a day prior to sensitization. On day 23 following HDM challenge, mice were exposed to serial challenges with 10, 20, 40 and 100 mg/ml aerosolized methacholine to measure lung dynamics. Furthermore, BALF and whole lung samples were harvested to examine pulmonary inflammation. Camel milk effectively inhibited both HDM-induced infiltration of eosinophils and AHR. In addition to this, camel milk downregulates the number of pulmonary Th2 and Th17 cells and suppressed CCL17 expression in whole lung homogenates. Furthermore, camel milk reduced HDM-induced IL-4 and IL-13 expression following *in vitro* restimulation of pulmonary T cell subsets. Additionally, camel milk suppressed total concentrations of IL-5 and IL-13 in the lung. These results corroborate the asthma-preventive potential of camel milk and highlight the significance of diminished local concentrations of Th2- associated cytokines. In the present study, the observed downregulation of asthma progression by camel milk suggests its potential health benefits; however, further experimental and controlled clinical trials are needed before it can be considered a supplementary approach for allergic asthma management.

## Introduction

Asthma, a widespread chronic inflammatory disease affecting up to 300 million individuals globally, is increasingly prevalent, especially in Western nations [1,2]. This ailment manifests through airway hyperresponsiveness, eosinophilic inflammation, and overproduction of mucus. These symptoms, including difficulty breathing, wheezing, coughing, and chest tightness, predominantly impact those with genetic predispositions to the disease.

A variety of environmental triggers such as airborne allergens, respiratory infections, and pollutants can cause asthma episodes [3]. The notable rise in asthma and allergy cases in Western nations recently is often attributed to the decline in rural living conditions, alongside dietary and lifestyle changes [4]. This phenomenon, known as the “hygiene hypothesis,” is supported by several experimental studies indicating that the likelihood of development of asthma and allergies significantly lower in individuals who grew up on farms [5–7].

Globally, camel milk has been widely utilized for its therapeutic and preventive capabilities in treating various conditions such as asthma, cancer [8], diabetes [9], and immune disorders [10,11]. Camel milk (CM), distinct from other mammalian milks, is richer in minerals, vitamins, and antioxidants [12–14]. Compared to cow milk, it contains higher levels of whey proteins such as lactoferrin and IgG, along with other antibodies [15,16]. The correlation between IgG deficiency and the development of asthma and allergies is well established [17,18]. Given its high IgG content, camel milk presents as a viable option for children with asthma and food allergies [19,20]. Additionally, the inflammatory nature of asthma is a relevant factor in this context [21]. Based on available literature, camel milk is noted for its ability to reduce levels of pro-inflammatory cytokines such as tumor necrosis factor (TNF), transforming growth factor beta (TGF-β), interleukin 6 (IL-6), and interleukin 17 (IL-17) [22,23]. Consequently, it may be effective in alleviating symptoms associated with certain diseases.

Given the rising use of camel milk and its pharmacological properties, this study was designed to investigate camel milk’s ability to prevent asthma development using a mouse model induced by house dust mite (HDM) allergens.

## Materials and methods

### Experimental design

#### Mouse model of asthma induced by house dust mite intranasal challenge.

BALB/c mice were purchased from the Charles River Laboratories. Female mice aged 8- to 10-weeks-old (catalog number: BALB/cAnNCrl). were maintained in a specific pathogen–free facility at the Marat Ospanov Medical University, with a 12-hour light cycle. Mice were provided with a standard rodent chow diet containing 3.5% fat, and 6.5% crude fiber, 15% protein, supplemented with a vitamins/minerals mixture, along with water ad libitum. Mice were divided into three groups, 5 mice per group: Naive, Camel milk+ HDM (CM/HDM) and HDM only. A previously established mouse model of asthma was used, in which mice were sensitized with HDM (Greer Laboratories, USA) following the protocol described by Waldstein, Issimov [24]. In brief, BALB/c mice were intranasally sensitized with vehicle or HDM in 2.5 µl (5 µg) per nostril, 5 days a week for 3 weeks. Following HDM sensitization, on day 22, mice received an HDM challenge by a large volume but low dose, of HDM challenge into the lung (5 µg in 50µl) using intranasal administration. The experiment was run two times and mean values were calculated.

During the study, mice received 0.5 ml of camel milk or vehicle five times a week, starting a day prior to sensitization, through oral gavage. The camel milk used was obtained from healthy adult female dromedary camels (Camelus dromedarius) aged between 6 and 10 years, maintained at a licensed commercial dairy farm located in the Almaty region, Kazakhstan. The animals were kept under standard feeding and husbandry practices. All milk was collected during mid-lactation.

### Airway hyperreactivity

Mice were anesthetized with Ketamine, tracheostomized, and paralyzed with Rocuronium Bromide. Mice were then ventilated with a computer-controlled ventilator (FlexiVent, Scireq, Canada) operating at 300 breaths/min. The ventilation involved a tidal volume of 6 cc/kg and a PEEP of 3 cm. Baseline measurements of airway resistance and the responsiveness of the bronchial system to inhaled aerosolized methacholine were determined by serial challenges with 10, 20, 40 and 100 mg/ml methacholine (Catalog No: 1396364, Millipore) at 10-second intervals for 2 minute as previously described [25]. Physiological parameters included airway resistance (Raw), tissue damping (G), tissue elastance (H) and peripheral airway elastance (ERS).

### Bronchoalveolar lavage

On day 24, mice were sacrificed by cervical dislocation. Lungs were cannulated and lavaged with 1 ml of pyrogen-free saline at 37°C, enriched with a protease inhibitor (Complete Mini, Roche Diagnostics, Germany). This procedure was repeated three times. The collected bronchoalveolar lavage fluid (BALF) was centrifuged at 400g for 5 min, and cell pellets from all lavages were combined. Cell counts in BALF were determined using a CellDrop-BF-UNLTD (Brightfield, DeNovix, DE, USA). Differential cell counts were conducted on cytospin preparations stained with Diff-Quick, identifying lymphocytes, macrophages, eosinophils and neutrophils via compound microscope. A minimum of 200 cells were counted to calculate the absolute number of each cell type.

### Lung restimulation

In vitro lung restimulation using HDM allergen was conducted by preparing single-lung cell suspensions. Lung samples were minced and incubated with a digestion buffer containing 125 U/ml collagenase (Catalog No:17100017, Sigma-Aldrich) and 60 U/ml DNase I (Catalog No: 11284932001, Sigma-Aldrich). The process was halted with fetal bovine serum, and the tissue was then strained and washed with 4 ml RPMI (Catalog No: 11-875-101, Gibco). Next, cells were rinsed and resuspended using RPMI 1640 medium, supplemented with antibiotics (10 U/ml penicillin, 10 μg/ml streptomycin sulfate) and inactivated fetal bovine serum. Cultured lung cells (3 × 10 ^5^ cell/well) were exposed to either a medium alone or with house dust mite allergen. After four days of culture, the supernatant was collected and stored at – 80°C for subsequent analysis.

### Flow cytometry

Following euthanasia, whole lungs were harvested and digested for 30 minutes at 37°C in 4 ml of Hank’s Balanced Salt Solution (Catalog No: 14025092, Gibco) supplemented with 125 U/ml collagenase (Catalog No:17100017, Sigma-Aldrich) and 60 U/ml DNase I (Catalog No: 11284932001, Sigma-Aldrich). GentleMACS C tubes (Miltenyi) were used to homogenize lungs in a GentleMACS Octo Dissociator per lung homogenization protocols. Lung homogenates were strained through a 70 µm nylon filter to create single cell suspensions. For intracellular staining, 2 x 10 ^6^ cells were plated in RPMI 1640 supplemented with 10% FCS (Catalog No: N-0500-A, Atlanta Biologicals), 5 nM 2-mercaptoethanol (Catalog No: 63689, Sigma-Adrich), 2 mM L-glutamine (Catalog No: 25030081, Gibco), 10 U/ml penicillin/streptomycin sulfate (Catalog No: 15140122, Gibco), 10 mM HEPES (Catalog No: 15630080, Gibco), 1mM Sodium Pyruvate (Catalog No: 11360070, Gibco), and 0.1 mM MEM non-essential amino acids (Catalog No: 11140050, Gibco). Cells were stimulated with PMA (50 ng/mL) and 500 ng/ml ionomycin (Catalog No: 501783946, Sigma-Adrich) and 10 μg/ml brefeldin A (Catalog No: 00-4506-51, BD Biosciences.) for 5 hours at 37°C. After stimulation, cells were harvested, fixed, and permeabilized using a fixation/permeabilization kit (BD Cytofix/Cytoperm). Fluorochrome-conjugated antibodies against intracellular targets were then added and incubated in the dark at 4°C. After washing, cells were analyzed using a flow cytometer (e.g., BD LSRFortessa). Fluorochrome-conjugated antibodies against surface markers (e.g., CD4) and intracellular targets. Samples were run on a Cytek Aurora flow cytometer and data were processed using FlowJo software (Tree Star Inc). Antibodies list in Table 1.

**Table 1:** Monoclonal antibodies used for flow cytometry.

*Antibodies*	*Source*
CD4-PerCP Cy5.5	Catalog No: 561115, BD Biosciences, Clone: SK3
GATA3-PE	Catalog No: 560074, BD Pharmingen, Clone: L50-823
Tbet-eFluor®660	Catalog No: 50-5825-82, eBioscience, Clone: eBio4B10
RORγt-PE	Catalog No: 562607, BD Pharmingen, Clone: Q31-378
CD25-Alexa Fluor®488	Catalog No: 53-0252-82, eBioscience, Clone: eBio7D4
FoxP3-APC	Catalog No: 17-4776-42, eBioscience, Clone: PCH101
CD11c-PerCP Cy5.5	Catalog No: 117328, Biolegend, Clone: N418
MHCII-PE Cy7	Catalog No: 60–5321, Cytek Biosciences, Clone: M5/114.15.2
CD11b-PE	Catalog No: 101207, Biolegend Clone: M1/70

### ELISA

Concentrations of CCL17, CCL20, CCL22, and IL-33 in lung tissue were determined using the DuoSet ELISA kit, while IL-5 and IL-13 levels were determined via Ready-SET-Go!® ELISA. Additionally, cytokine levels in lung restimulation supernatants were assessed using standard IL-13 flex set and T1/T2/T17 assays. The experiments were carried out according to the manufacturer’s instruction.

### Statistics

Graphs were compiled and statistical analyses were conducted using Prism 9 software (GraphPad Software, Inc., San Diego, CA). The data underwent evaluation through a variety of statistical tests, including Student’s t-test and one-way ANOVA with Tukey’s post hoc analysis. Outlier detection was performed using the ROUT method with a Q value of 1%. The assumptions of normality and variance were verified prior to applying parametric tests. All results are presented as the mean ± standard error of the mean (SEM) from two independent experiments (n = 5 per group per experiment, total n = 10 per group). Statistical significance between groups was denoted when P < 0.05 using asterisks, as detailed in the respective figures.

## Results

### Camel milk inhibits HDM induced airway hyperresponsiveness and inflammation in the lungs

**Fig 1:**
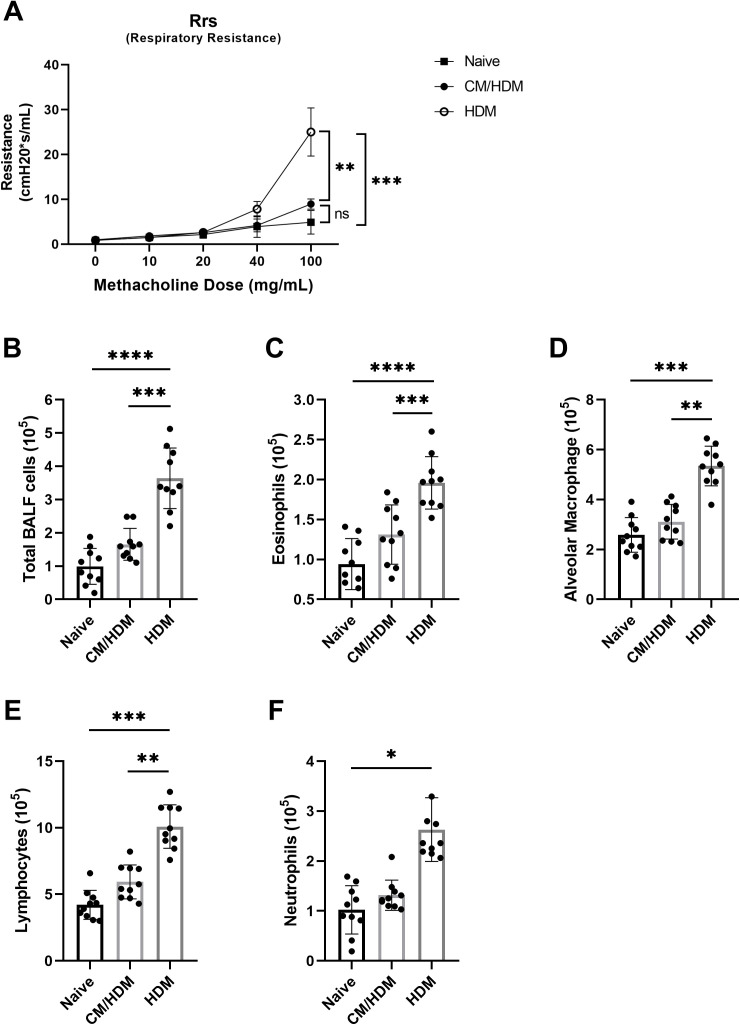
Camel milk abolishes increased AHR and lung inflammation in mice with HDM-induced asthma. BALB/c mice were treated with HDM (2.5 μl per nostril, 25 µg/5µl in total) or vehicle 5 days a week for 4 weeks. Methacholine induced changes in resistance of airway Rrs (Respiratory system resistance) were measured using FlexiVent instrument (A). From bronchoalveolar lavage fluid, a total BALF cells (B), eosinophils (C), alveolar macrophages (D) lymphocytes (E), neutrophils (F) were counted using cytospin with Diff-Quic staining. Data presented as the mean ±SEM from two combined independent experiments (n = 5 per experiment). For statistical analysis, a one-way ANOVA with a Tukey’s multiple comparison test was performed. Asterisk over bars demonstrated level of significance, where *p < 0.05, **p < 0.01, ***P < 0.001, ****p < 0.0001.

To evaluate the impact of camel milk on pulmonary function, airway hyperresponsiveness (AHR) was quantified following the administration of various doses of methacholine. Baseline measurements of AHR in HDM exposed mice were elevated compared to those treated with vehicle. HDM exposed mice group demonstrated an increased AHR following methacholine challenge as opposed to vehicle treated counterparts (Fig 1A).

To investigate the magnitude of airway inflammation, BALF was analyzed. A significant elevation in the total inflammatory cell count in the BALF of HDM-exposed mice was observed compared to naïve and CM/HDM mice (Fig 1B). This increase was predominantly attributed to elevated number of eosinophils (Fig 1C). Additionally, there were also increased levels of macrophages, lymphocytes and neutrophils (Fig 1D–F). Dietary supplementation of camel milk consumption led to a reduction in the total inflammatory cell count in the BALF, as evidenced by decreased numbers of eosinophils, macrophages, lymphocytes and neutrophils (Fig 1B–F).

### The effect of camel milk on inflamatory dendritic cells concentration in the lungs of allergen-challenged mice

**Fig 2:**
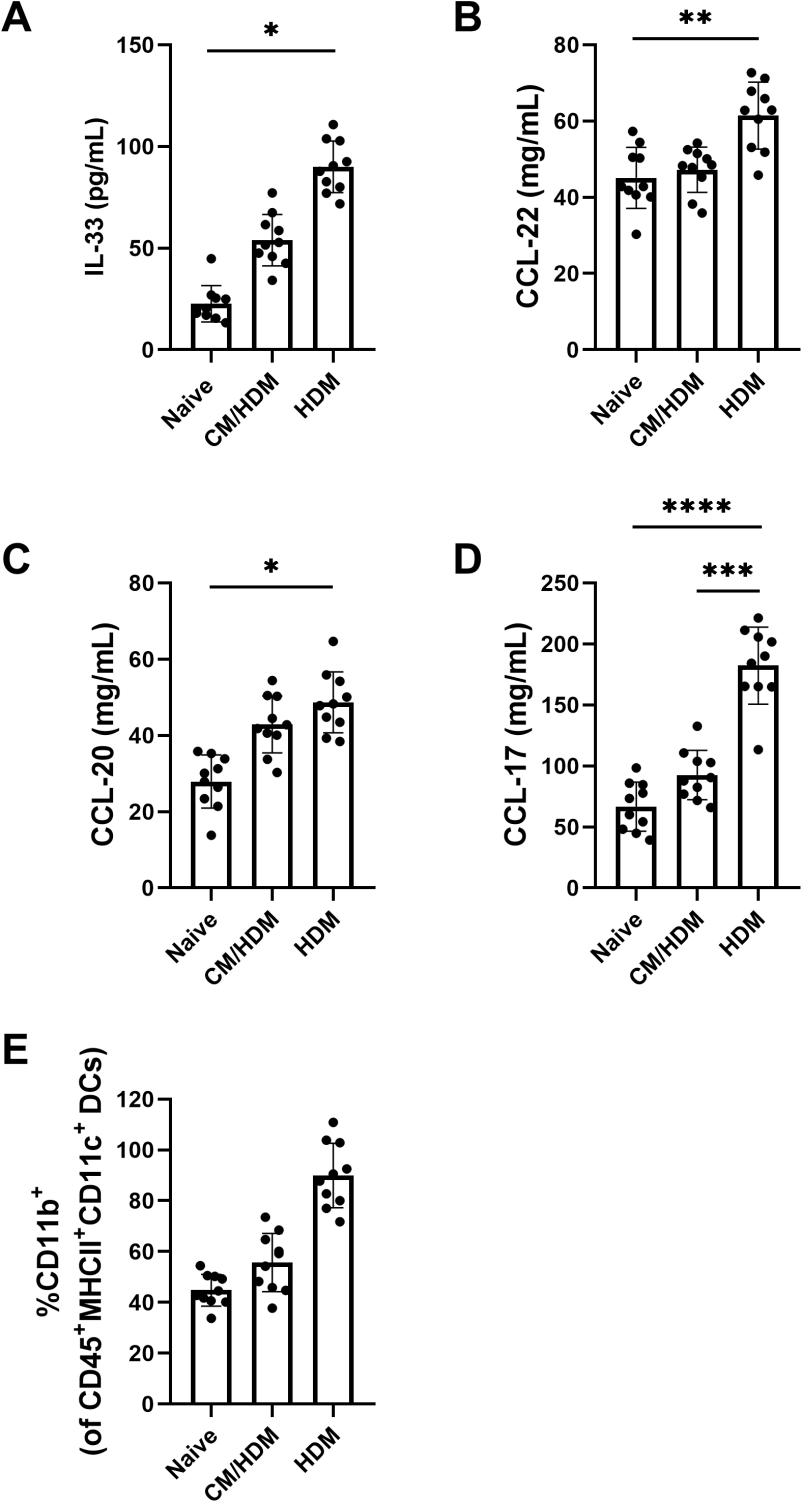
The number of CD11b + cDCs and the associated concentrations of CCL17 in the lung were diminished following exposure to camel milk. The concentrations of IL-33 (A), CCL22 (B), CCL20 (C) and CCL17 (D) were measured in whole lung homogenates using ELISA kit. The proportion of CD11b+cDCs was measured in whole lung suspension (E) S1 Fig. Data presented as the mean ±SEM from two combined independent experiments (n = 5 per experiment). For statistical analysis, a one-way ANOVA with a Tukey’s multiple comparison test was performed. Asterisk over bars demonstrated level of significance, where *p < 0.05, **p < 0.01, ***P < 0.001, ****p < 0.0001.

To define how camel milk can affect on pulmonary chemokines and cytokines, supernatants obtained from lung homogenates were examined. Concentrations of IL-33, CCL22, CCL20 and CCL17 were found to be elevated in HDM exposed mice relative to naive and CM/HDM mice group (Fig 2A–D). Camel milk had no effect on the concentrations of CCL20, CCL22 and IL-33 (Fig 2A–C), however it significantly reduced the concentration of CCL17 (Fig 2D). Given that CCL17 is predominantly expressed by CD11b + DCs [26], this specific inflammatory DC subtype in the lung suspensions was analyzed. An increased proportion of CD11b + DCs was detected in HDM-mice in comparison to naive and CM/HDM mice (Fig 2E).

### Camel milk reduces the number of Th2 and Th17 Cells recruited in the lungs following HDM exposure

**Fig 3:**
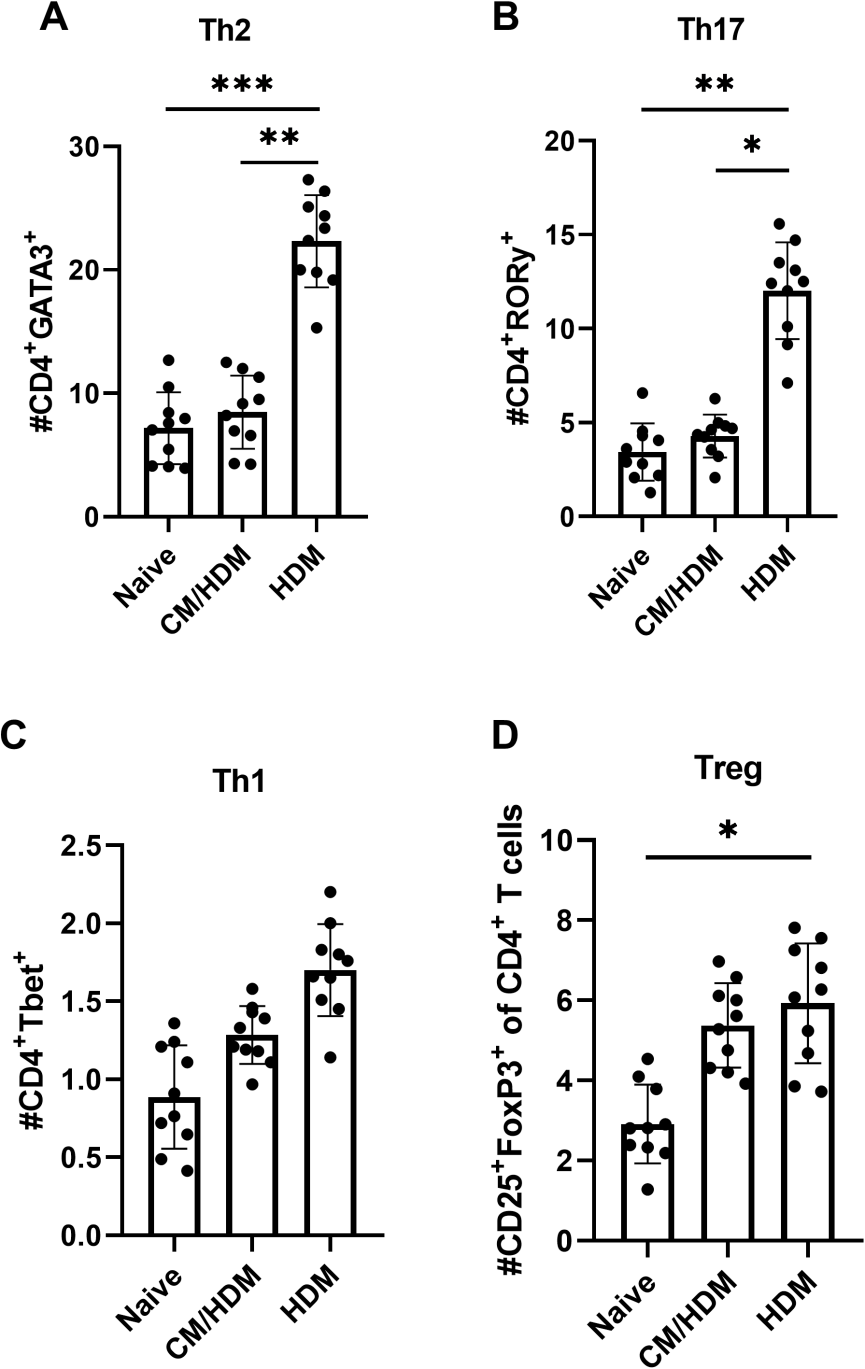
Camel milk reduced infiltration of Th2 and Th17 cells into the lungs. Whole lung homogenates were harvested and the total numbers of Th2 (A), Th17 (B), Th1 (C), T regulatory cells was determined by flow cytometry S2 Fig. Data presented as the mean ±SEM from two combined independent experiments (n = 5 per experiment). For statistical analysis, a one-way ANOVA with a Tukey’s multiple comparison test was performed. Asterisk over bars demonstrated level of significance, where *p < 0.05, **p < 0.01, ***P < 0.001, ****p < 0.0001.

Analyses were conducted on lung cell suspensions to evaluate T cell subsets. An elevated number of Th2, Th17, T regulatory cells was observed in HDM-exposed mice compared to CM/HDM and naive (Fig 3A–D). Corresponding with the decreased levels of the Th2- polarizing CCL17 chemokine, the prevalence of Th2 cells in the lung compartments was diminished by the administration of camel milk (Fig 3A). Similar tendency were detected for Th17 cells (Fig 3B), whereas the number of Treg cells were not affected by camel milk administration (Fig 3D). Likewise T1 cells were slightly elevated in HDM only treated mice compared to naïve and CM/HDM mice group (Fig 3C).

### Camel milk inhibits Th2 and Th17 associated cytokine release following in vitro lung restimulation using HDM

**Fig 4:**
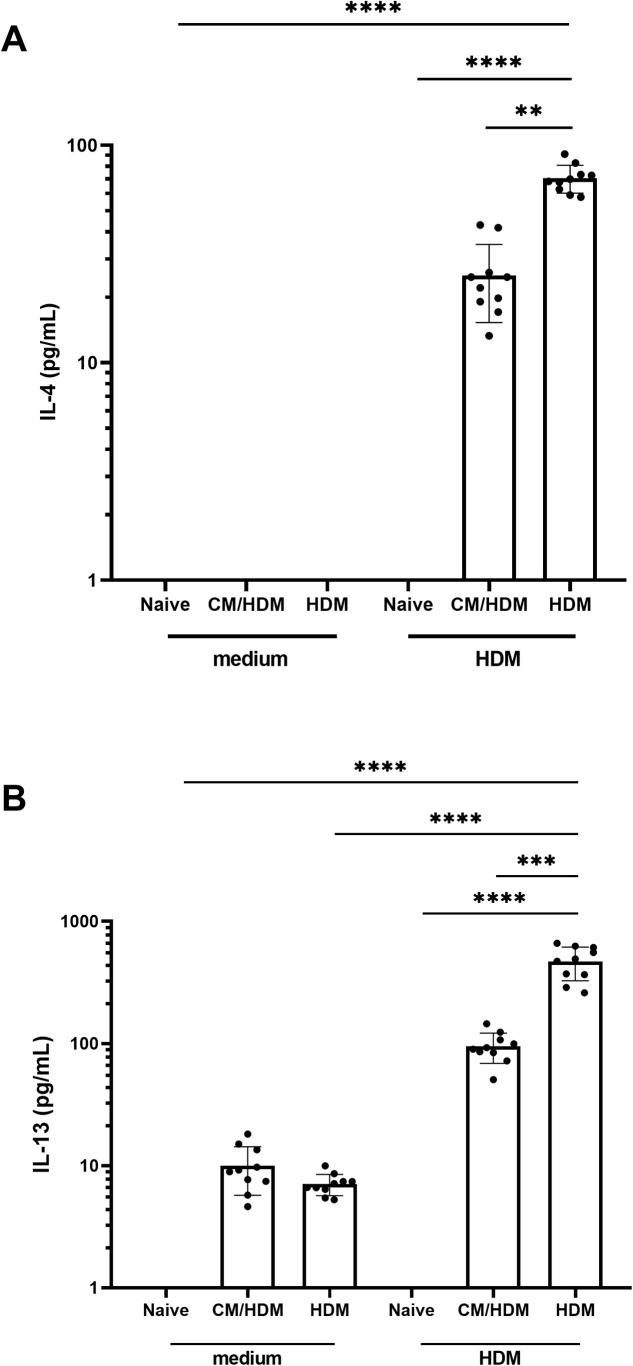
IL-4 and IL-13 expression following in vitro stimulation of whole lung cell suspensions with HDM was abolished by camel milk. Mice were challenged with HDM into the lungs (5 µg in 50µl) by intranasal administration, whole lungs were harvested and homogenised. Whole lung homogenates were then restimulated with medium or HDM for 4 days. The concentrations of IL-4 and IL-13 were analyzed in the supernatants. Data presented as the mean ±SEM from two combined independent experiments (n = 5 per experiment). For statistical analysis, a one-way ANOVA with a Tukey’s multiple comparison test was performed. Asterisk over bars demonstrated level of significance, where *p < 0.05, **p < 0.01, ***P < 0.001, ****p < 0.0001.

To determine whether the decrease in the number of Th2 and Th17 cells also influenced the synthesis of Th2 and Th17-associated cytokines, the presence of IL-4, IL-5, IL-13, and IL-17A in lung cell supernatants was quantified following in vitro restimulation using HDM. Th2 specific cytokine synthesis was not detected in vehicle mice, whereas HDM only exposed mice exhibited a significant augmentation in IL-4, IL-5 and IL-13 levels (Fig 4A, B). Camel milk significantly reduced the concentrations of IL-4, IL-5 and IL-13 in lung cell supernatants (Fig 4A, B). Despite an elevation in IL-17A levels in HDM only exposed mice in comparison to vehicle group, camel milk did not affect on IL-17A concentrations.

### *Camel milk inhibits* IL-4, IL-5 and IL-*13 cytokine responses following in vitro pulmonary challenge*

**Fig 5:**
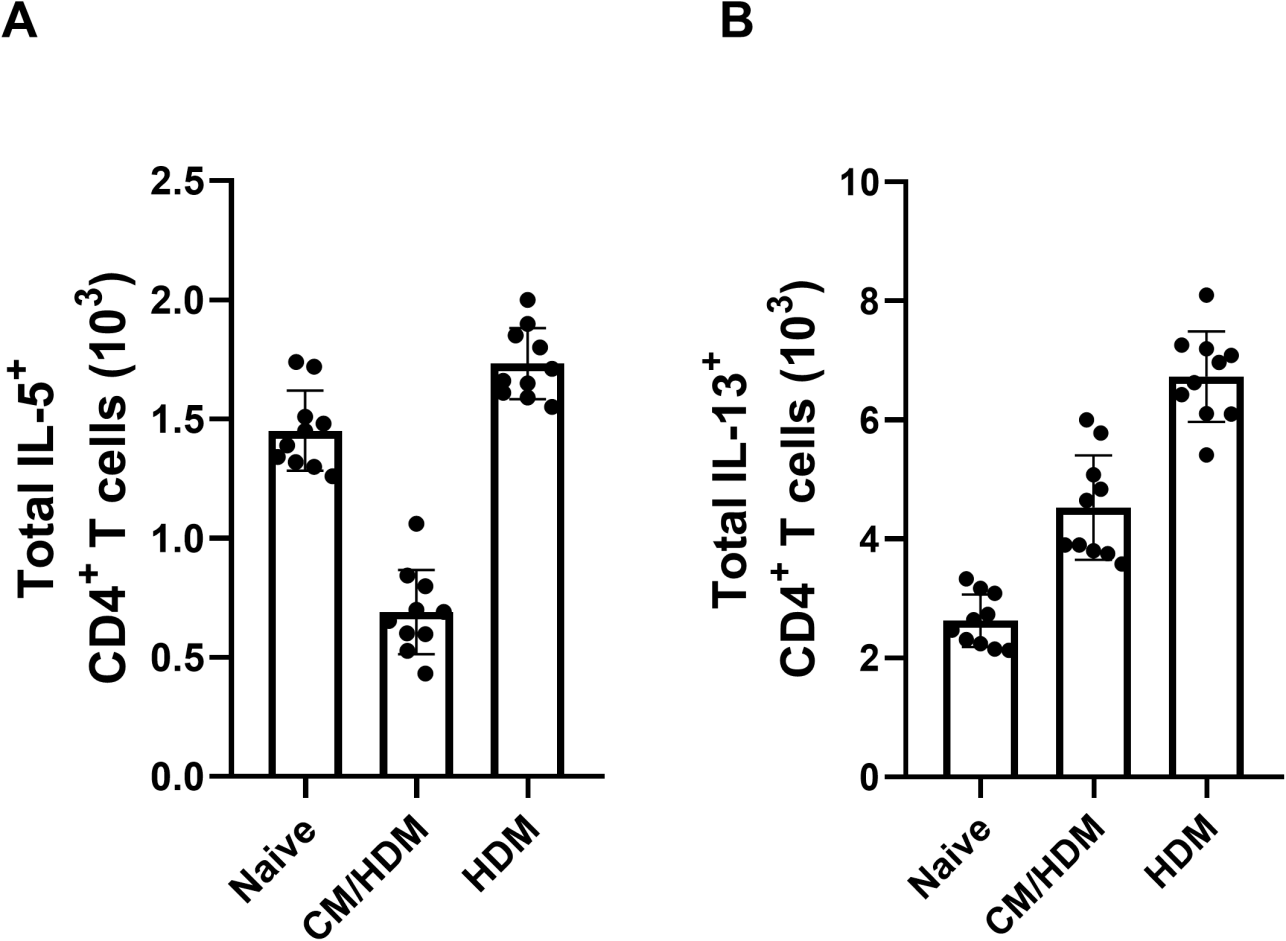
Camel milk suppressed the concentrations of IL-5 and IL-13 in the lung. The total concentration of IL-5 (A) and IL-13 (B) was determined by intracellular cytokine staining. Data presented as the mean ±SEM from two combined independent experiments (n = 5 per experiment). For statistical analysis, a one-way ANOVA with a Tukey’s multiple comparison test was performed. Asterisk over bars demonstrated level of significance, where *p < 0.05, **p < 0.01, ***P < 0.001, ****p < 0.0001.

To evaluate whether camel milk reduced Th2-associated cytokines in the lungs following HDM challenge, we analyzed supernatants from lung homogenates. IL-5 concentrations did not differ between HDM-exposed and naïve mice, whereas IL-13 levels were significantly increased in the HDM-only group (Fig 5A, B). Camel milk mitigated this elevation in IL-13 (Fig 5B), concurrently appearing to suppress IL-5 concentrations (Fig 5A).

## Discussion

In this study, we found that the intake of camel milk inhibited the onset of allergic asthma in a mice model following systemic exposure to HDM allergen. Robust protective effects were evident in terms of AHR and inflammation, aligning with an attenuated type 2 immune response. Cross-sectional studies conducted among European young populations have revealed that children raised in farms are less susceptible to develop asthma [6,7,27]. The findings of these studies provide insight into how exposure to farm-related environments can exert a protective effect against the development of systemic allergy or asthma. In recent years, several studies have been published on the health protective effects of consuming camel milk [10,11,23]. Collectively, these studies outline the observed associations and do not establish a causal relationship between camel milk consumption and asthma prevention. Although our study demonstrates that camel milk exerts protective effects against HDM-induced asthma, we acknowledge that the specific bioactive constituents responsible for these effects were not directly measured S1 Table. This model imitates a cardinal feature of asthma and possesses clinical significance owing to the utilization of HDM allergen in human pulmonary exposure [28–30]. Others demonstrated that camel milk possess therapeutic properties in preventing allergic asthma. Camel milk inhibited AHR associated with HDM exposure and abolished lung inflammation. While literature suggests that camel milk is rich in immunomodulatory components such as IgG, lactoferrin, and bioactive peptides [31,32], we did not perform constituent-specific quantification or fractionation in this study.

Fat content of raw cow milk is another component found to reduce risk of asthma development [33,34]. Given the similarity in fat content between cow milk (3.7%) and camel milk (3.5%), we speculate that the protective effect observed against asthma in our study may be partially attributed to the fat content of camel milk. Although we did not experimentally assess cow milk in this study, previous research has demonstrated that raw cow milk consumption is associated with a reduced risk of asthma and allergic diseases in humans [35–37]. In our study, we observed that camel milk significantly reduced airway hyperresponsiveness and levels of Th2-associated cytokines in a murine model of asthma. These findings are consistent with previous research indicating that camel milk possesses immunomodulatory and anti-inflammatory properties. For example, a study by Kruzel, Bacsi [38,39] found that lactoferrin suppresses pollen-induced allergic airway inflammation in a murine model of asthma using BALB/c mice. They hypothesize that lactoferrin reduced the ragweed-induced increase in cellular reactive oxygen species (ROS) levels in bronchial epithelial cells through its iron-binding properties.

Additionally, a study by Bakhtiari, Nekouhi [10] supports our findings by illustrating how dietary interventions, including the consumption of milk, can influence Th2 immune responses and modulate asthma symptoms. However, unlike our results, some studies have shown varying degrees of efficacy, which may be attributed to differences in experimental design, including the type of model used (e.g., ovalbumin vs. HDM) [40]. Moreover, it is important to note that camel milk composition may vary depending on geographic origin, camel breed, lactation stage, and environmental factors. In the present study, milk was sourced from a single farm to minimize variability; however, interregional and batch-to-batch differences could influence the concentrations of bioactive components [41,42].

To elucidate the mechanisms through which camel milk confers asthma-protective effects, we examined various inflammatory mediators implicated in asthma pathogenesis. HDM is a common allergen that triggers an activation of epithelial cells in allergic asthma [43]. Activation of epithelial cells occurs when HDM allergens and Toll-like receptor binds on epithelial membrane. Upon activation, these cells express cytokines and chemokines, which attract DCs to the lung and orchestrate allergic inflammation. Together, this process involving innate immune function of airway epithelial cells entails the induction of type 2 immune response [44]. As dendritic cells themselves express pattern recognition receptors (PRRs), they can undergo direct activation in response to HDM allergens [1]. In our model, mediators derived from both epithelial cells and DCs (Fig 2) were elevated following HDM exposure. Likewise the number of CD11b+ conventional DCs were increased. These findings align with human studies that demonstrate elevated levels of CCL17, CCL20, CCL22, and IL-33, as well as an increased number of DCs, in the airways of asthmatic individuals compared to control groups [45–47]. Epithelial mediators were not upregulated following camel milk intake, while the concentration of CCL20 and the number of CD11b + cDCs were downregulated. In this case, the subpopulation of cDC plays a crucial role in promoting allergic inflammation through the expression of CCL22 and CCL17 [48]. These chemokines act as chemoattractant ligands for CCR4 and facilitating the selective migration of Th2 cells expressing CCR4 to the lungs, implying the onset of allergic inflammation [49]. The significance of CCL22 and CCL17 is further affirmed in murine models of asthma, where the utilization of specific neutralising antibodies enables the reduction of airway hyperresponsiveness and allergic inflammation [50,51].

The decrease in the concentration of CCL17 derived from CD11b+ conventional dendritic cells, resulting from the intake of camel milk, might have contributed to a reduction in the influx of Th2 cells. In fact, the presence of GATA3 + CD4 + Th2 cells in the lung was diminished by the intake of camel milk. It is known that GATA3 expression is significantly elevated in T cells obtained from airway biopsies of individuals with asthma compared to those from healthy controls [52]. GATA3 is crucial for the secretion of allergen-specific Th2 cytokines and plays a pivotal role in the differentiation of naive T cells into Th2 cells [53,54]. More specifically, the intake of camel milk has been demonstrated to decrease the quantity of Th17 cells, in addition to reducing the number of Th2 cells in the lung. These Th17 cells primarily activate the upregulation of neutrophils in individuals with asthma [55]. Indeed, this decrease in Th17 cell is aligned with a reduction in the influx of neutrophils into the lung. Whilst the precise role of Th17 cells in HDM-associated allergic asthma is yet to be fully understood, there is growing interest in the therapeutic potential of inhibiting Th17 immune response [56,57]. In addition, camel milk suppressed the production of Th2-associated allergen-specific IL-4, IL-5 and IL-13 cytokines upon in vitro lung T cells restimulation with HDM. Collectively, these cytokines contribute to the prominent characteristics of Th2‑driven allergic asthma [58].

This study is the first to evaluate the effects of camel milk on house dust mite (HDM)-induced asthma in a murine model. The valuable findings of the present study indicate that camel milk effectively inhibited both HDM-induced eosinophils infiltration and airway hyperresponsiveness (AHR). Furthermore, camel milk downregulated the number of pulmonary Th2 and Th17 cells and suppressed the expression of CCL17 in whole lung homogenates. Additionally, camel milk reduced HDM-induced expression of IL-4 and IL-13 in pulmonary T cell subsets and suppressed the total concentrations of IL-5 and IL-13 in the lung. Our findings underscore the novel therapeutic potential of camel milk in managing allergic asthma, highlighting its immunomodulatory and anti-inflammatory properties through the reduction of local concentrations of Th2-associated cytokines. Although camel milk has been clinically associated with improved asthma outcomes, including in a 2019 human trial [59], the immunological mechanisms underlying its effects remain unclear. Our study extends existing knowledge by employing a BALB/c HDM-induced asthma model to uncover specific pathways modulated by camel milk. We demonstrate that camel milk suppresses airway hyperresponsiveness, Th2 and Th17 cell recruitment, and associated cytokines (IL-4, IL-5, IL-13, IL-17A), while reducing dendritic cell activity and CCL17 expression in the lungs. These findings suggest that camel milk disrupts allergen-driven immune activation at both innate and adaptive levels. This mechanistic understanding will help define the pharmacological properties and therapeutic potential of camel milk as part of allergen-specific immunotherapy in asthmatic patients.

This study has several limitations that should be acknowledged. First, although camel milk is known to contain bioactive components such as IgG, lactoferrin, and various peptides with immunomodulatory properties, we did not perform fractionation or quantify individual constituents in the present work. As such, we cannot attribute the observed effects to specific components. Second, we did not include a direct comparison with cow milk, which is more widely consumed and has also been reported to modulate immune responses. While our primary objective was to characterize the protective effects of camel milk on HDM-induced asthma and its underlying immunological mechanisms, future studies will be designed to incorporate cow milk as a comparator to enhance translational relevance and generalizability of our findings.

## Supporting information

S1 Table Biomolecular Content of Camel Milk. (PDF)

S1 Fig Gating strategy for associated FACS plots. (TIF)

S2 Fig Gating strategy for Th2, Th17, Th1, T regulatory cells. (TIF)
