# Supplementary material for: The effect of camel milk on house dust mite allergen induced asthma model in BALB/C mice
Source: PLoS One. 2025 Jun 27;20(6):e0327504. doi: 10.1371/journal.pone.0327504 (PMC12204568; doi:10.1371/journal.pone.0327504)
Supplement: S1 Table — (PDF) [file pone.0327504.s001.pdf]

Supplementary Table: *Biomolecular Content of Camel Milk*

The data presented in this table summarize the biomolecular composition of the camel milk used in this study. Values in the **"Component"** and **"Approximate Concentration"** columns represent quantitative measurements obtained from pooled pasteurized camel milk samples used in our experiments. These values were determined through laboratory analyses conducted in duplicate to ensure accuracy and reproducibility. The **"Notes"** column provides additional context drawn from previously published literature to support interpretation and comparative evaluation of the measured components. These references are cited to highlight known biological functions.

Component	Approximate Concentration	Notes
Total protein	3.3%	Slightly lower than cow milk*
Fat	4%	High in long-chain unsaturated fatty acids**
Lactoferrin	0.3 mg/mL	High antibacterial, antiviral, anti-inflammatory activities**
IgG	7.5 mg/mL	Major immunoglobulin; contributes to immune defense*
Lysozyme	0.3 mg/mL	Antibacterial enzyme, higher than in cow milk***
Vitamin C	47 mg/L	3–5 times higher than in cow milk*
Caseins	60% of total protein	Lower allergenicity compared to cow milk**
Whey proteins	20% of total protein	Rich in protective bioactive peptides**

* Konuspayeva, G. S. (2020). Camel milk composition and nutritional value *Handbook of Research on Health and Environmental Benefits of Camel Products* (pp. 15-40): IGI global.

** Khalesi, M., et al., *Biomolecular content of camel milk: A traditional superfood towards future healthcare industry.* Trends in Food Science & Technology, 2017. **62**: p. 49-58.

*** Seifu, E. (2023). Camel milk products: innovations, limitations and opportunities. *Food Production Processing and Nutrition, 5*(1). doi:10.1186/s43014-023-00130-7
