# Supplementary figures and images for: The effect of camel milk on house dust mite allergen induced asthma model in BALB/C mice
Source: PLoS One. 2025 Jun 27;20(6):e0327504. doi: 10.1371/journal.pone.0327504 (PMC12204568; doi:10.1371/journal.pone.0327504)

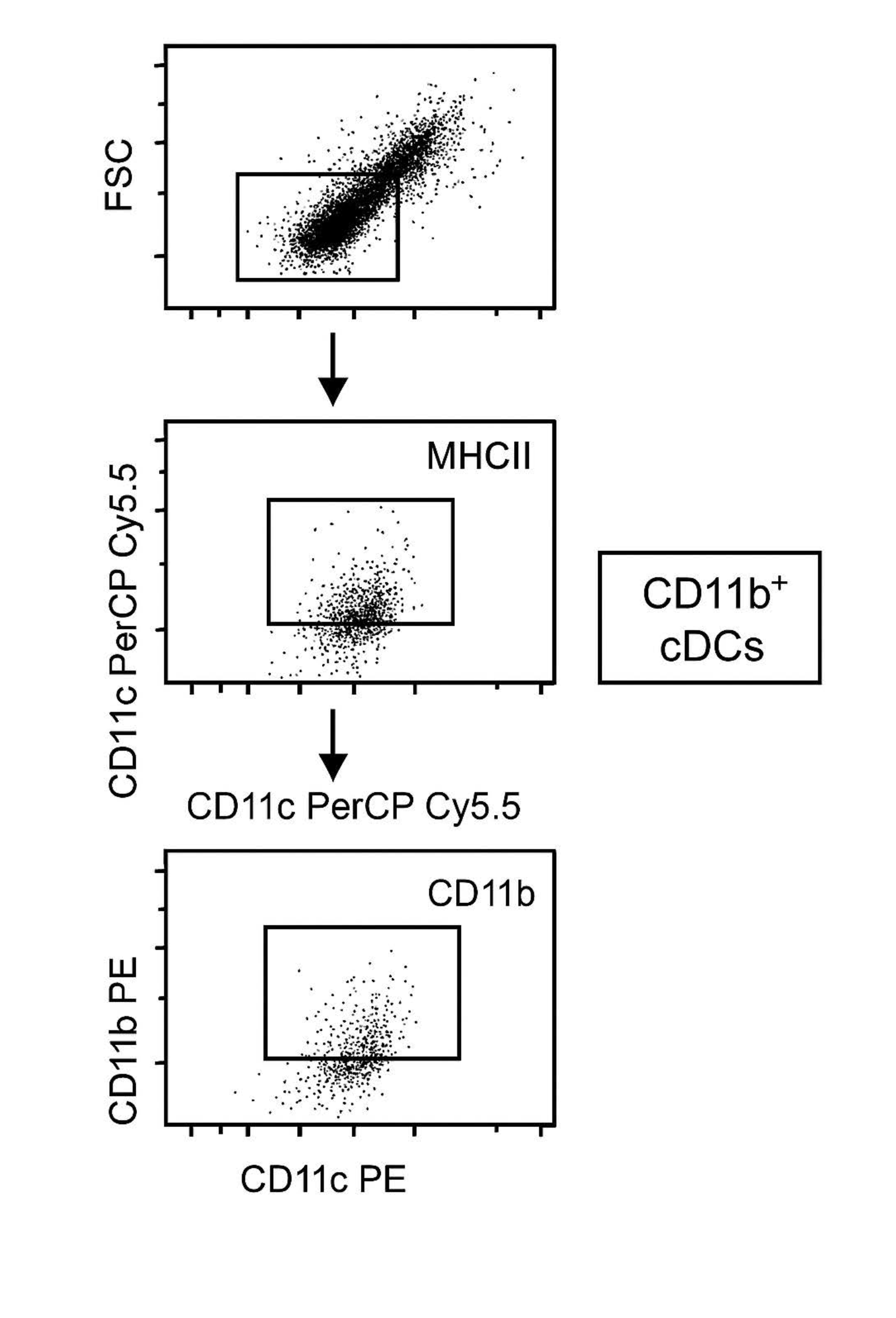

Supplement: S1 Fig — (TIF) [file pone.0327504.s002.tif]

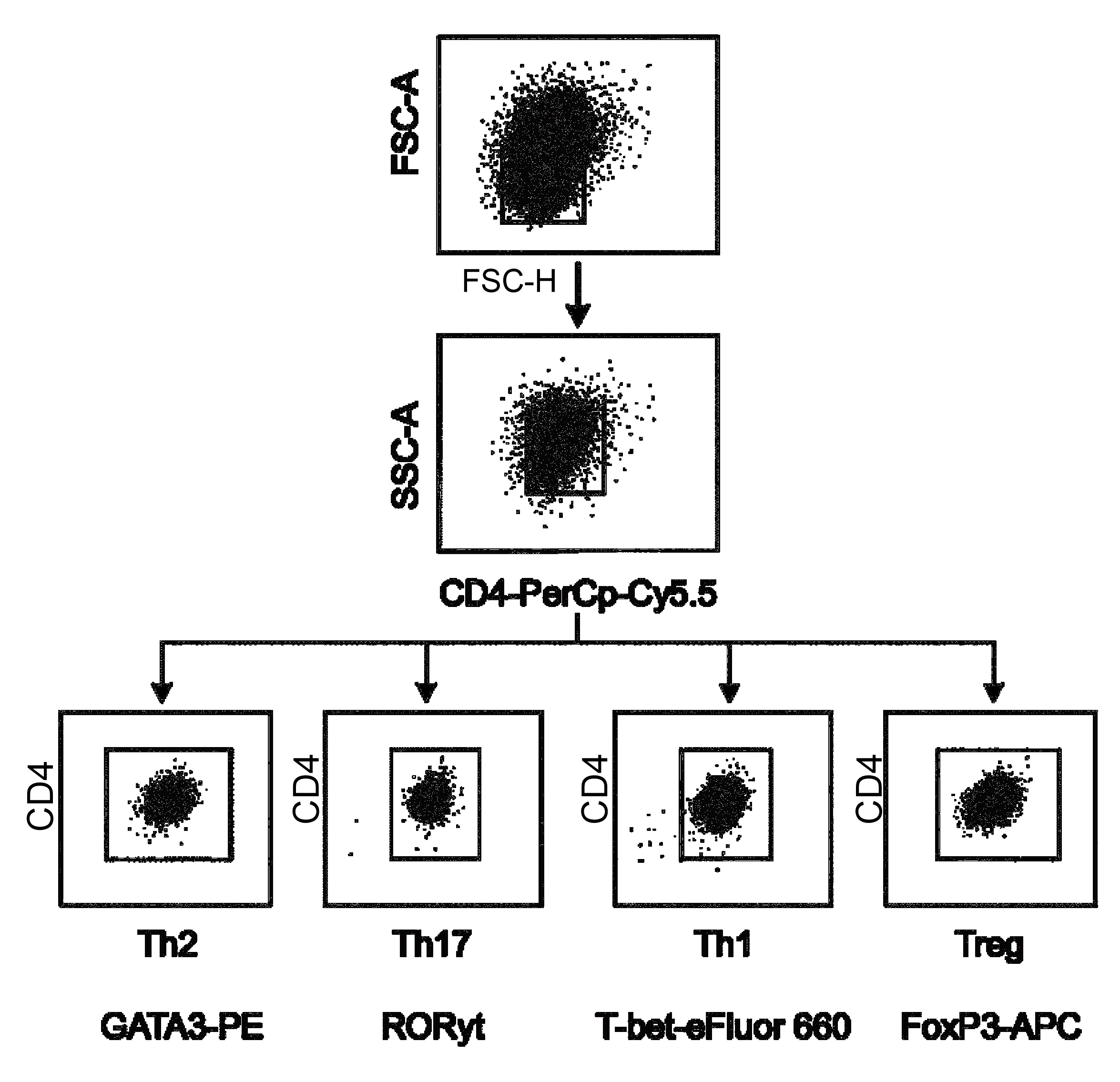

Supplement: S2 Fig — (TIF) [file pone.0327504.s003.tif]
